# San Bernardino County Youth Opioid Response: Improving Access to Evidence-Based Medical Treatment for Opioid Use Disorder

**DOI:** 10.7759/cureus.9781

**Published:** 2020-08-16

**Authors:** Michael M Neeki, Fanglong Dong, Benjamin Archambeau, Melinda Cerda, Sireyia Ratliff, Alan Goff, Kristina Roloff, Louis Tran

**Affiliations:** 1 Emergency Medicine, Arrowhead Regional Medical Center, Colton, USA; 2 Probation Department, San Bernardino County Department of Probation, San Bernardino, USA; 3 Emergency Medicine, California University of Science and Medicine, Colton, USA; 4 Obstetrics and Gynecology, Arrowhead Regional Medical Center, Colton, USA; 5 Obstetrics and Gynecology, California University of Science and Medicine, Colton, USA

**Keywords:** medication-assisted treatment, opioid use disorder, youth, adolescence, harm reduction, buprenorphine

## Abstract

Opioid use disorder (OUD) and related overdose deaths have become a crisis of epidemic proportions in the United States. In 2018, over 10 million people age 12 years or older misused opioids.Substance use is also correlated with increased physical and mental health disorders, and developmental challenges among youths. Medication-assisted treatment (MAT) has been reported to reduce mortality, opioid use, and human immunodeficiency virus and hepatitis C virus transmission while increasing treatment retention in adults. The San Bernardino County Youth Opioid Response (SBCYOR) program was formed to explore best practices for youths at risk of opioid use disorders and/or overdose. SBCYOR is a coalition of professionals in healthcare, behavioral health, public education, law enforcement, emergency medical services (EMS) agencies, and juvenile detention centers throughout San Bernardino County, California. SBCYOR focuses on high-risk and addicted individuals between the ages of 12 to 24 years in San Bernardino County’s correctional system. It utilizes a strategy of collaboration, prevention, risk mitigation, medication, psychological treatment, and community outreach. This study aimed to evaluate the implementation and progress of SBCYOR.

## Introduction

The rate of opioid use and dependency has increased to the point of being a major public health priority in the United States over the last decade. In 2018, the National Survey on Drug Use and Health (NSDUH) reported that 10.3 million people aged 12 years or older have misused opioids [1]. The associated medical and social complications related to opioid use disorders (OUDs) have also grown proportionately. Addiction disorders strain public resources, emergency departments (EDs), and correctional institutions. In 2018, California providers issued more than 19.8 million prescriptions for opiates despite the 2,428 documented opiate-related deaths [2]. The opioid addiction epidemic continues to pose an ongoing threat to the health of Americans.

The youth populations (aged 12-24 years) are particularly vulnerable and have also been affected by the opioid crisis. Youths may lack prefrontal cortical function leading to behavior inhibition and risk aversion [3]. This contributes to a more substantial addiction pathology. In 2017, more than 70,000 died from drug overdoses, making it the leading cause of injury-related deaths in adolescents and young adults in the United States [4]. Youths also have more serious mental illnesses, depression, and suicidality with a strong correlation to substance abuse [5]. High risk sexual behaviors in this population also lead to sexually transmitted infections and/or unintended pregnancy. Almost nine in 10 pregnancies in adults with OUD are unintended, and it is prudent to anticipate similar prevalence among youths [6].

The office of the Surgeon General has stated that medication-assisted treatment (MAT) combined with psychosocial therapy and community-based recovery support is the gold standard for treating opioid addiction [7]. However, youths may be excluded from these addiction treatment programs, as many programs are geared toward adults. Feder et al. reported that only 2.4% of adolescents received MAT, as compared to 26.3% of adults [8]. Additionally, youths may experience greater barriers in accessing care such as lack of insurance, reliable transportation and parental notification concerns [9]. These barriers are magnified in youths with low socioeconomic status, in part due to complexities in health insurance coverage and access to safe treatment for drug dependence. In 2017, Orgera and Tolbert suggested that patients with Medicaid coverage were more than 2.5 times likely to receive treatment for OUD than uninsured patients [10]. Even among commercially insured youths, only 25% received MAT for OUD [11].

Treatment strategies for OUD are complex and multifactorial. Recent emphasis on MAT in conjunction with cognitive behavioral therapy (CBT) has shown to be effective in treating OUD in adults [12]. Although not well studied among adolescents and young adults, clinical experience has not identified any age-specific safety concerns. Matson et al. demonstrated that continued outpatient therapy with buprenorphine/naloxone (Suboxone) achieved a high rate of long-term sobriety among adolescents and young adults [13]. The American Academy of Pediatrics has recommended that pediatricians consider MAT for adolescents with OUD [14].

The San Bernardino County Youth Opioid Response (SBCYOR) program was formed to help mitigate the spread of OUD, increase access to MAT and reduce the risk of overdose for the youth populations. The program is a part of the California Youth Opioid Response (YOR California) Project. SBCYOR is a coalition of professionals in healthcare, behavioral health, public education, law enforcement, emergency medical services (EMS) agencies, and juvenile detention centers throughout the county. It focuses on high-risk and addicted individuals between the ages of 12 to 24 years in San Bernardino County’s correctional system. The program utilizes a strategy of collaboration, prevention, risk mitigation, medication, psychological treatment, and community outreach. Its goal is to integrate the county’s available resources into a continuum of care to enable the youths of the community to grow into productive members of society, free of the burden of opioid use exposure, disorder and associated risks. The objective of this study is to evaluate the implementation and sustainability of the SBCYOR program and its effects in the community to date.

## Materials and methods

This is a prospective observational study of youths aged 12 to 24 years identified through San Bernardino County’s correctional, educational, and healthcare systems. Data were extracted from the electronic health records (EHR) of the county’s Department of Probation, the Sheriff’s Department, and Arrowhead Regional Medical Center (ARMC), a county owned and operated safety net hospital. San Bernardino County (SBC) is the largest geographic county in the United States with a diverse population of 2.2 million people. According to the 2018 Census data, the racial makeup of SBC was 52.3% Latino, 29.8% White, and 8.0% African American [15]. The county has a large proportion of low socioeconomic status individuals. Approximately 15% of the population lives below the poverty level, and more than a quarter of the population is younger than 18 years old [16].

Patients enrolled in the program were tracked and results from data were reported to YOR California, in compliance with state and federal regulations. This study was approved by the ARMC Institutional Review Board. Eligibility for inclusion in this study was between age 12 to 24 years with a reported history of opioid use, the presence of opioid on a screening drug test, physical signs of drug use, or evidence of overdose or withdrawal. Physical signs of drug use included the presence of track marks, lethargy, flu-like symptoms, unexplained somnolence or respiratory depression. A Clinical Opiate Withdrawal Scale (COWS) score was used to assess the degree of opioid withdrawal.

Exclusion criteria was age less than 12 or greater than 24 years, refusal of enrollment, hypersensitivity to recommended medication, inability to obtain consent, active withdrawal from alcohol or benzodiazepine, or medical frailty. Medical frailty was determined by an attending emergency physician in consultation with other subspecialties, after a thorough evaluation of the patient’s medical history and present illness. Examples of medical fragility include decompensated heart failure, severe liver disease, or poorly controlled psychosis.

The program required participating providers to obtain an X waiver. The X waivered providers received extensive training and education in key aspects of MAT including identification of individuals who may benefit from treatment, safe and appropriate use of Federal Drug Administration-approved medications, strategies for communicating and educating patients and their families, and coordinating care with other community infrastructure.

SBCYOR implemented its community outreach program in phases (Table 1) with regular analysis and modifications based on efficacy measures. Within each stage, there were overlapping action items that spanned over the entire timeline of the program.

**Table 1:** Summary of timeline for SBCYOR development SBCYOR: San Bernardino County Youth Opioid Response; MAT: Medication-assisted treatment; OUD: Opioid use disorder.

STAGE	ITEM	ACTION
Definition of goals	Decrease morbidity and mortality of target population	Use of naloxone, identify and enroll applicable youth in MAT programs, build connection with community resources
	Increase access to effective treatment	Establish treatment protocols, safe prescribing practices, increase x-waiver providers
	Decrease stigma associated with OUD	Educate affected youth and their family, participate in community outreach
	Improve continuity of care	Implement warm handoff of patients, assign a substance use navigator to coordinate care
Planning & development	Analysis of the current system	Identification of effective leadership
	Identification of key partner organizations	Collaborate with providers, detention centers, behavioral health, drug courts, school district, and local law enforcement agencies
	Identification of rate limiting steps	Consider HIPAA compliance and sharing data among the various participating agencies, de-identify personal information in accordance with 42 CFR part 2 regulations
Establish coalition	A cohesive group of community organizations to address multifactorial treatment in environments for care	Involve county and community hospitals, SBC schools, Sheriff Department, Department of Probation and Department of Behavioral Health
Implementation	Procedures and protocols for screening, referral, treatment, pregnant patients, and connection to additional resources	Initiate screening programs and treatment protocols in the detention centers for those self-reported, suspected, or identified with OUD within target group
	Division of tasks	Determine site-specific responsibilities among coalition members, providers, nursing staff and custody staff
	Statistics capturing and reporting system	Develop a process to capture meaningful statistical data
	X-waiver certified providers	Ease enrollment and incentivize to increase provider participation
	OUD/MAT training	Conduct initial and ongoing training for providers and health staff
Outcome measures	Measurement and monitoring of outcomes	Establish weekly review of outcome measurements by program director and steering committee along with monthly and quarterly statistical reporting
Sustainability	Gain financial independence	Organizational restructuring to integrate into current funding systems
	Obtain community support	Provide evidence of positive outcomes and solicit feedback, offer access to information regarding program capabilities
	Maintain regular communication channels	Create a website and social media outlets for accessibility and community engagement

Phase 1: Definition of goals

After a thorough situation analysis, SBCYOR agreed to focus on four primary objectives to guide design and implementation of the program. These four primary objectives were:

To decrease the morbidity and mortality in the target population

To increase access to effective treatment including medication assisted treatment

To decrease the stigma associated with OUD within the community

To improve continuity of care for the target population

Phase 2: Planning & development

To achieve these goals, SBCYOR reached out and connected with the community, healthcare providers, and county agencies. Team members attended community events, gathered and shared information and resources, and provided education for families to recognize addiction as a chronic medical condition, the signs and symptoms of OUD. Emphasis was placed on reducing exposure to opioid use and prevention of overdose. Because OUD in youth can begin with appropriately prescribed opiates, health care providers were educated about risk mitigation for patients and household members. Mitigation efforts also included education and access to naloxone (the reversal agent for an opiate overdose). The coalition also explored the formation of a referral network, connecting patients to the existing resources for behavioral counseling services and MAT within the community and the county.

SBCYOR established treatment protocols and safe prescribing practices amongst youths with OUD, allowing X waivered providers to initiate MAT by prescribing buprenorphine/naloxone. The X waivered providers also assessed youths at risk for OUD, and performed interventions deemed appropriate. Leadership of the program connected with established OUD treatment programs such as the California Bridge (CA-BRIDGE) project, an initiative which developed hospitals’ emergency departments into primary access points for treating acute symptoms of OUD. SBCYOR integrated with CA-BRIDGE’s established network, coordinated by ARMC, to help transition youths to outpatient-based MAT clinics for continued therapy.

Phase 3: Establishing coalition

SBCYOR collaborated with local and county agencies to form a coalition. These agencies included school district leadership, law enforcement, EMS, correctional facilities, behavioral health centers, and hospitals. Specifically, SBCYOR connected with the county’s Department of Probation, Department of Behavioral Health, the Sheriff’s Department, ARMC, and the Inland Empire Opioid Crisis Coalition (IEOCC). Site champions within each organization were identified and enrolled into the effort. Once the coalition was established, these agencies were invited to actively participate in planning, refining, and eventual implementation of the program. SBCYOR also reached out to community resources such as the local Harm Reduction Coalition and MAT clinics of the California Hub and Spoke System (CA H&SS) who also became key partners.

Phase 4: Implementation

**Figure 1:**
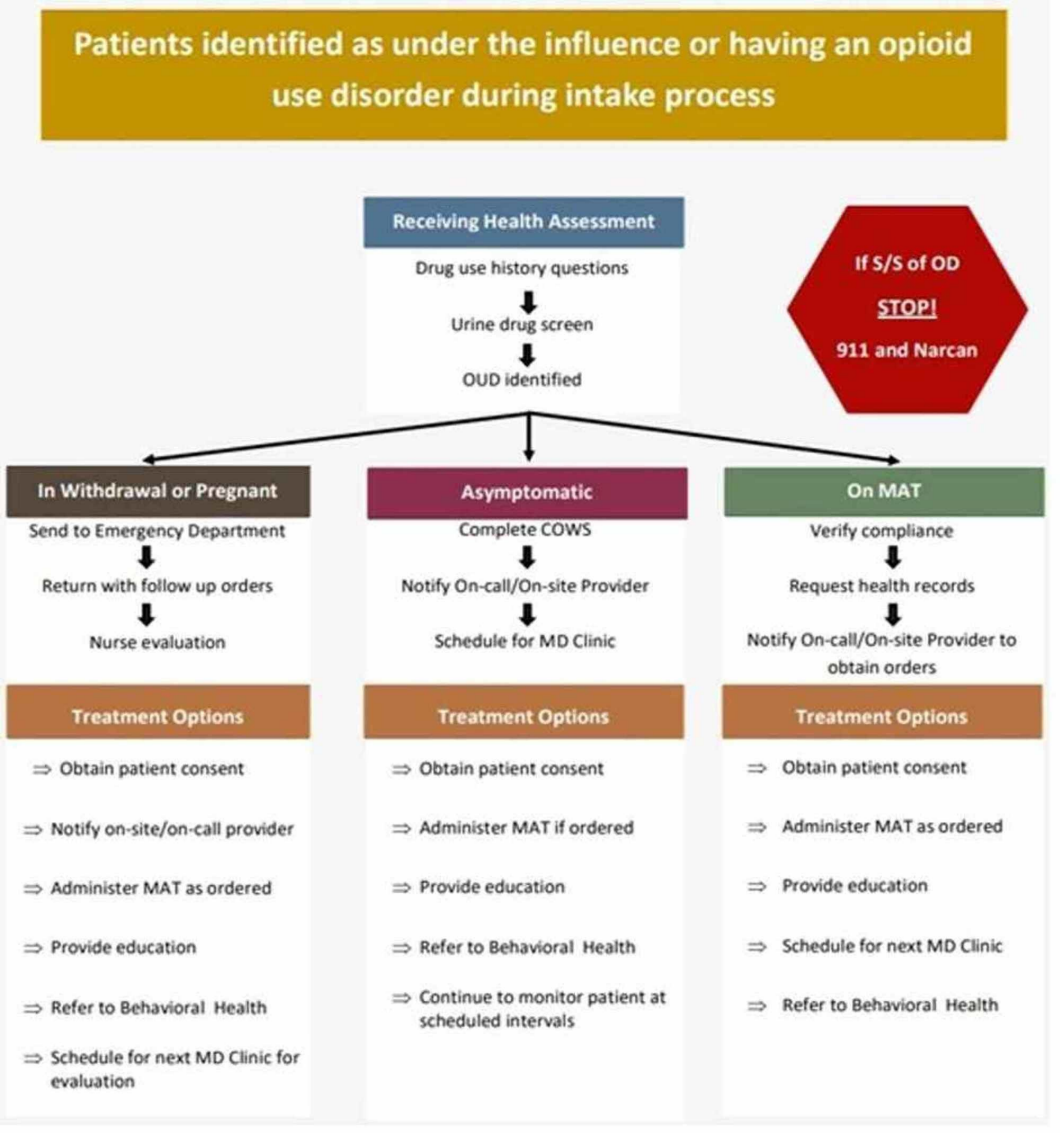
Triage algorithm

The program officially launched on August 1, 2019. The coalition provided site-specific training and technical assistance for healthcare providers in the detention facilities that would have encounters with the target population. Screening assessment tools were created for nursing staff in the detention centers to streamline the process for appropriate referrals and treatment. Figure 1 presents the detailed triage algorithms. These screening assessment tools were instrumental in identifying and enrolling individuals who would potentially benefit from the program. SBCYOR facilitated access to emergency departments and outpatient treatment centers for MAT therapy. In addition, the program has an ongoing series of community outreach initiatives, including public service announcements, community events, and on-site teaching to increase and maintain community awareness and involvement. The program has also embraced technology in its efforts with the use of remote health screening and social media.

Designated case managers and substance use navigators (SUNs) helped guide patients through the complexities of the healthcare system, addressing their specific needs. They connected patients to outpatient MAT centers based on specific needs such as insurance coverage, transportation, and appropriate referrals for comorbid conditions. The SUN is expected to play a significant role in a patient’s recovery, facilitating social support to help with living situations and dealing with external stressors. Most importantly, the SUN provided the patient with a constant point of contact whenever needed.

Phase 5: Outcome measures

All eligible youths identified by the county health staff within the Probation and Sheriff’s Departments detention centers were tracked. Similarly, information on all youths who received MAT through Arrowhead Regional Medical Center (ARMC) were collected. Data collection was reported by providers and tabulated by the SUN. Providers were able to notify the SUN through phone calls, HIPAA compliant emails, or the consult function of the electronic record. Once the patient was released from a facility, the SUN followed the youth via individualized home visits, phone calls or video telehealth visits.

Data were collected and reviewed weekly by the program director and steering committee. A more detailed analysis of the compiled data was performed monthly and quarterly. Based on the observations and findings of these analyses, the program was continually refined accordingly. The goal was to identify areas of need for immediate attention and/or resources diversion.

Phase 6: Sustainability

While the SBCYOR program was created through grant funding from the Substance Abuse and Mental Health Services Administration response to the opioid epidemic, it was important to institutionalize practices and partnerships to gain financial independence with the goal of becoming less reliant on future grant funds. Thus, the program focused on embedding best practices and workflows at an organizational level to be integrated into funding systems of each coalition partner. The MAT program in each institution is now integrated into the standard medical services provided by physicians and advanced providers and has become part of standard practice.

## Results

After initial preparation and coordination with the grant originating organization, California Institute for Behavioral Health Solutions (CIBHS), implementation of the SBCYOR program was initiated in August 2019. The core operations team was formed and members were assigned roles and responsibilities. A timeline was created with goals and milestones established. The core team set out to secure site champions at contributory facilities. These facilities included California University of Science and Medicine (CUSM) and its affiliated hospital, ARMC, along with West Valley Detention Center, SBC Juvenile Detention Center, and local school districts as target sites. A website and online social media presence were created to announce the launch of the program and give a point of contact for the community.

Over the next three months, policies and procedures were created and the medical staff at participating institutions were trained. The program implementation was piloted in the juvenile detention center and later expanded to the adult detention center. By October 2019, 90% of the healthcare staff in the detention centers received training on OUD and MAT. The educational team members also reached out to train other county’s facilities and programs, such as halfway houses, public schools, and law enforcement agencies.

In December 2019, an agreement with local Probation leadership was reached to include the opioid use disorder and naloxone (Narcan) training into the permanent training protocol for the probation academy program. The program trains 150-300 new officers per year. Narcan training was incorporated into standard departmental training for all officers, with 550 additional officers trained per year. In order to reach the largest audience, SBCYOR is working with the local school districts to bring the educational teams to junior high and high schools.

**Figure 2:**
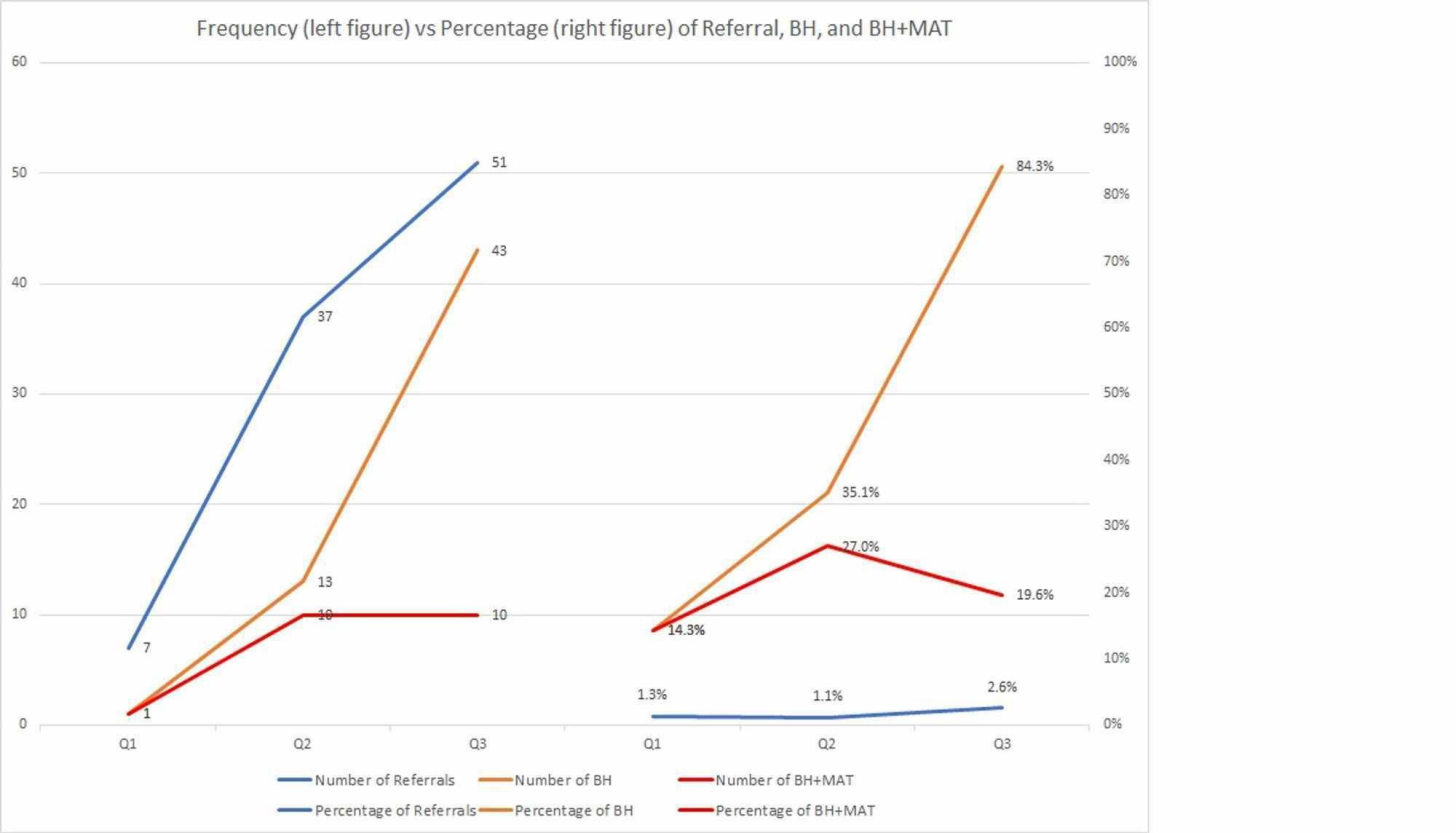
Frequencies and percentage of patients referred for evaluation and patients receiving therapy

In the first quarter (Q1: from Aug 1, 2019 - Nov 30, 2019), the program screened 520 youths, and seven (1.3%) of those screened were referred for evaluation into YOR services. Of the first seven referred, one (14.3%) youth met criteria for treatment by Behavioral Health (BH). The same youth also received MAT therapy. Enrollment increased in the second quarter (Q2: Dec 1, 2019 - Feb 29, 2020), with 3244 youths screened and 37 (1.1%) referred for treatment. Among those who were referred, 13 (35.1%) youths received BH, and 10 (27%) youths received both MAT and BH. In the third quarter (Q3: Mar 1, 2019 - May 31, 2020), 1929 youths were screened, with 51 (2.6%) of them referred. Among those who were referred, 43 (84.3%) youths received BH, and 10 (19.6%) youths received MAT and BH. The total number of patients referred for evaluation (51; 2.6%) and received treatment (43; 84.3%) both increased from Q2 to Q3. In total, 60% (57/90) of patients referred for evaluation were successfully enrolled into treatment. Table 2 and Figure 2 present the detailed summary and graph presentation.

**Table 2:** Summary of patients referred for evaluation and subsequent treatment by quarters BH: Behavioral health; MAT: Medication-assisted treatment.

Quarter*	Patients screened	Referred for evaluation	Treated with BH**	Treated with BH & MAT***	% Screened referred for eval	% Referred treated with BH	% Referred treated with MAT & BH
Q1	520	7	1	1	1.3%	14.3%	14.3%
Q2	3244	37	13	10	1.1%	35.1%	27.0%
Q3	1,929	51	43	10	2.6%	84.3%	19.6%

The decrease in total screens from Q2 to Q3 is thought to be a consequence of the current COVID-19 pandemic. During Q3, California Governor Gavin Newsome issued stay-at-home orders to mitigate the spread of the SARS-CoV-2, the coronavirus that is responsible for COVID-19. The crime rates in SBC saw a subsequent drop when compared to 2019 [17]. This likely affected the number of individuals being sent to correctional facilities. Despite the decrease in total youths screened, the total number of patients referred for evaluation (51; 2.6%) and treatment (43; 84.3%) both increased from Q2 to Q3.

Data collection became more detailed as the program grew and developed. By Q3, SBCYOR was able to tabulate and evaluate demographic data for all patients who were enrolled into the system. Table 3 presents detailed demographic data. Among the 1929 youths who were screened, a significant majority were males (71.6%, n = 1381) and in the 18-24 years age group (89.6%, n = 1729). More than half were Hispanic or Latino (51.2%, n = 987), followed by Black or African American (29.2%, n = 564) and White (17.7%, n = 342). Despite preparation for treatment of pregnant youths within this program, we did not receive a referral for pregnancy during the study period.

**Table 3:** Demographic summary of youth in Q3 (Mar 1, 2019 - May 31, 2019). Complete demographic data was not available in Q1 & Q2.

	N = 1,929	Percent
Gender		
Female	548	28.4%
Male	1381	71.6%
Race		
White	342	17.7%
Black or African American	564	29.2%
Hispanic or Latino	987	51.2%
Native Hawaiian or Other Pacific Islander	4	0.2%
Asian	15	0.8%
Other	17	0.9%
Age		
12 - 17	200	10.4%
18 - 24	1729	89.6%

## Discussion

Opioid use disorders are chronic conditions with substantial personal and societal costs [18]. The pathophysiology of opioid use disorder is well understood. Morgan and Christie have demonstrated that opioid tolerance may develop rapidly, even after limited use [19]. Susceptible individuals will therefore experience more pain and discomfort than normal when the opioid effect is removed. This can lead to recreational misuse, and addiction in people who use opioid medications chronically [19].

Substance use disorder is a medical condition with structural and chemical brain changes [7]. Dugosh et al. have concluded that behavioral therapy alone is not effective in maintaining abstinence in patients with OUD [20]. Medication-assisted treatment (MAT) has been effective and is currently the gold standard for treating OUD [7]. Current clinical evidence indicates that the risk of relapsing increased when pharmacotherapy is discontinued. Therefore, treatment should be individualized to each patient without a set time for discontinuation of pharmacotherapy [21, 22].

Subramaniam et al. demonstrated that youths suffering from OUD commonly have multiple comorbidities, including polysubstance abuse, psychiatric disorders, hepatitis C infection, a history of high-risk sexual and criminal behaviors [23]. They are at high risk for sexually transmitted infections and unintended pregnancies. Pregnancy in women with OUDs is associated with adverse outcomes such as fetal growth restriction, preterm labor, stillbirth, and neonatal abstinence syndrome (NAS) [24]. These patients are best managed by a multidisciplinary team that utilizes MAT and additional counseling and preparation for NAS [25]. Unfortunately, pregnant women referred from criminal justice agencies for treatment of OUDs receive evidence-based therapies less often than those referred from other sources [26]. SBCYOR aims to reduce health care disparities among pregnant youths in SBC with OUD by including maternal fetal medicine physicians as part of the collaborative team. As a result, integrated contraceptive services, access to long-acting reversible contraceptive methods, and access to abortion services as well as comprehensive longitudinal care for the continued pregnancy were incorporated into the SBCYOR program to ensure comprehensive treatment and health equity amongst participants.

Opioid use disorder also places a significant burden of cost on society affecting health care, social service, and justice systems. A strong effort must be made to target this group early with both preventative and therapeutic options in order to mitigate downstream effects. People affected by OUD are often ashamed of their problem. They fear social stigma associated with drug use, and implications with law enforcement involvement [27]. This problem may be amplified in the youth population because of relative social and cognitive immaturity. As a result, many at risk or affected youths are not identified until they have downstream complications such as an overdose and/or incarceration. By then, addiction may have already profoundly and negatively impacted their lives. It is a challenge to encourage youths who are in the earlier stages of opioid dependence to self-identify and seek treatment. Therefore, future directions of the SBCYOR program may include screening for at-risk youths at all public education institutions. To this end, SBCYOR is currently engaging the San Bernardino School District in a pilot project to identify opportunities for collaboration.

Historically, substance use disorder treatment of any kind, nationally and statewide, has been offered under the Substance Abuse Prevention and Treatment Block Grant. Multiple public systems have responded to growing community needs resulting in diverse and uncoordinated approaches. Discrete system and programming responses has resulted in an inability to share data. This lack of data sharing has been a significant barrier to forming a coordinated effort in developing a continuity of care for these patients. Previous efforts at forming a centralized database in San Bernardino County for all partners have been limited, and often lacking vital elements of the patient’s health history, such as medication, dosage, and response to treatment. This adversely impacts the delivery of care as the patient moves along the spectrum of recovery [28, 29]. While confidentiality regulations present challenges, these can be overcome with interagency cooperation and patient informed consent. The response to the opioid crisis must be broad in its scope in order to be effective. It must incorporate medication therapy, evidence-based practices, and solutions to psychosocial components unique to each individual [30]. SBCYOR plans to enroll its coalition partners in developing a centralized and HIPAA compliant regional data sharing system, which will allow a multidisciplinary team to properly develop and coordinate an effective care plan.

This crisis affects more than just the individuals who misuse opioid medications and illicit drugs. Repercussions of this crisis reverberate throughout the entire community. The stigma surrounding OUD and deficits in current treatment strategies must be addressed. Among the youth population, OUD is a growing concern. Any strategy will need to understand the systemic barriers and be sensitive to unique attributes of this population. Without a cultural transformation of the system, no recovery program can be successful long-term. Actions to address the crisis requires the endorsement from the community and coordination from multiple agencies and organizations. Without active support from the county organizations, public schools, law enforcement agencies, local fire departments, social services and department of behavioral health, it would be impossible to reach affected youths and provide them with essential and ongoing care. As such, SBCYOR has successfully brought together community stakeholders, especially public service entities on the frontlines of the epidemic. Together, community partners can disseminate information, provide support, offer an alternative to drug use, and participate in therapeutic interventions. Consideration must be given to local, state, and federal regulations to this socially sensitive field of medicine.

The greatest challenge moving forward for continued implementation of programs such as SBCYOR is securing the commitment of agencies to the implementation of best practices. Procuring on-going funding is necessary; however, much can be achieved by system, organizational, practitioner commitment to change. There has been significant financial, government, and community support to initiate this campaign, but to this point, further funding beyond the first year of implementation has not been secured. Additionally, in light of a new challenge in response to the COVID-19 pandemic, the coalition plans to utilize telemedicine and other available technologies in an effort to prevent delay in screening, care and treatment. Ultimately, the organizational will, innovations, and pathways must be established for continued funding without dependence on grants. This may be achieved by refining the logistics, bringing more OUD awareness, and government and community buy-in.

## Conclusions

Opioid use disorder is a chronic condition with substantial health, economic and social costs. SBCYOR was created as a coalition to help fill a gap in the national effort to address the opioid use crisis in SBC. It specifically targets youths aged 12-24 years in SBC suffering from OUD. Early results of SBCYOR suggested that the coalition has enrolled youths in MAT programs and provided an integrated response, showing promising results, and has successfully partnered with community stakeholders. Future systematic implementation of various clinical and administrative integration strategies is needed in order to ensure a better continuum of care and success of this program.

## References

[1] Substance Abuse and Mental Health Services Administration (2020). Key Substance Use and Mental Health Indicators in the United States: Results from the 2018 National Survey on Drug Use and Health. Results from the.

[2] (2020). California opioid overdose surveillance dashboard. https://skylab.cdph.ca.gov/ODdash/.

[3] Váša F, Seidlitz J, Romero-Garcia R (2018). Adolescent tuning of association cortex in human structural brain networks. Cerebral Cortex.

[4] (2020). Drug overdose deaths in the United States, 1999-2017. https://www.cdc.gov/nchs/data/databriefs/db329-h.pdf.

[5] Subramaniam GA, Stitzer ML, Woody G, Fishman MJ, Kolodner K (2009). Clinical characteristics of treatment-seeking adolescents with opioid versus cannabis/alcohol use disorders. Drug Alcohol Dependence.

[6] Clayton HB, Bohm MK, Lowry R, Ashley C, Ethier KA (2019). Prescription opioid misuse associated with risk behaviors among adolescents. Am J Prev Med.

[7] U.S. Department of Health and Human Services (HHS), Office of the Surgeon General (2018). Facing Addiction in America: The Surgeon General’s Spotlight on Opioids. http://addiction.surgeongeneral.gov/sites/default/files/Spotlight-on-Opioids_09192018.pdf.

[8] Feder KA, Krawczyk N, Saloner B (2017). Medication-assisted treatment for adolescents in specialty treatment for opioid use disorder. J Adolesc Health.

[9] Chelvakumar G, Ford N, Kapa HM, Lange HL, McRee A-L, Bonny AE (2017). Healthcare barriers and utilization among adolescents and young adults accessing services for homeless and runaway youth. J Commun Health.

[10] Orgera K, Tolbert J (2019). The opioid epidemic and Medicaid’s role in facilitating access to treatment. Henry J Kaiser Family Foundation.

[11] Hadland SE, Wharam JF, Schuster MA, Zhang F, Samet JH, Larochelle MR (2017). Trends in receipt of buprenorphine and naltrexone for opioid use disorder among adolescents and young adults, 2001-2014. JAMA Pediatr.

[12] Srivastava A, Kahan M, Nader M (2017). Primary care management of opioid use disorders: abstinence, methadone, or buprenorphine-naloxone?. Can Fam Physician.

[13] Matson SC, Hobson G, Abdel-Rasoul M, Bonny AE (2014). A retrospective study of retention of opioid-dependent adolescents and young adults in an outpatient buprenorphine/naloxone clinic. J Addict Med.

[14] Committee on Substance Use and Prevention (2016). Medication-assisted treatment of adolescents with opioid use disorders. Pediatrics.

[15] (2020). Profile of the city of San Bernardino. https://www.scag.ca.gov/Documents/SanBernardino.pdf.

[16] (2020). QuickFacts, San Bernardino County, California. https://www.census.gov/quickfacts/sanbernardinocountycalifornia.

[17] (2020). Non-violent crime drops in San Bernardino during coronavirus lockdown. https://www.sbsun.com/2020/04/27/non-violent-crime-drops-in-san-bernardino-during-coronavirus-lockdown/.

[18] Chetty M, Kenworthy JJ, Langham S, Walker A, Dunlop WC (2017). A systematic review of health economic models of opioid agonist therapies in maintenance treatment of non-prescription opioid dependence. Addict Sci Clin Pract.

[19] Morgan MM, Christie MJ (2011). Analysis of opioid efficacy, tolerance, addiction and dependence from cell culture to human. Br J Pharmacol.

[20] Dugosh K, Abraham A, Seymour B, McLoyd K, Chalk M, Festinger D (2016). A systematic review on the use of psychosocial interventions in conjunction with medications for the treatment of opioid addiction. J Addict Med.

[21] (2020). Information about medication-assisted treatment (MAT). https://www.fda.gov/drugs/information-drug-class/information-about-medication-assisted-treatment-mat.

[22] Coffa D, Snyder H (2019). Opioid use disorder: medical treatment options. Am Fam Physician.

[23] Subramaniam GA, Fishman MJ, Woody G (2009). Treatment of opioid-dependent adolescents and young adults with buprenorphine. Curr Psychiatr Rep.

[24] Committee on Obstetric Practice (2017). Opioid use and opioid use disorder in pregnancy. Am Coll Obstet Gynecol.

[25] Hurley EA, Duello A, Finocchario-Kessler S, Goggin K, Stancil S, Winograd RP, Miller MK (2020). Expanding contraception access for women with opioid-use disorder: a qualitative study of opportunities and challenges (PREPRINT). Am J Health Promot.

[26] Winkelman TN, Ford BR, Shlafer RJ, McWilliams A, Admon LK, Patrick SW (2020). Medications for opioid use disorder among pregnant women referred by criminal justice agencies before and after Medicaid expansion: a retrospective study of admissions to treatment centers in the United States. PLoS Med.

[27] Kennedy-Hendricks A, Barry CL, Gollust SE, Ensminger ME, Chisolm MS, McGinty EE (2017). Social stigma toward persons with prescription opioid use disorder: associations with public support for punitive and public health-oriented policies. Psychiatr Serv.

[28] Fleury M-J, Perreault M, Grenier G, Imboua A, Brochu S (2016). Implementing key strategies for successful network integration in the Quebec substance-use disorders programme. Int J Integr Care.

[29] Greiner MV, Beal SJ, Dexheimer JW, Divekar P, Patel V, Hall ES (2019). Improving information sharing for youth in foster care. Pediatrics.

[30] Clarke N, Mun EY, Kelly S, White HR, Lynch K (2013). Treatment outcomes of a combined cognitive behavior therapy and pharmacotherapy for a sample of women with and without substance abuse histories on an acute psychiatric unit: do therapeutic alliance and motivation matter?. Am J Addict.

